# T-bet expression in CD8+ T cells associated with chronic hepatitis B virus infection

**DOI:** 10.1186/s12985-016-0473-y

**Published:** 2016-01-25

**Authors:** Rongshan Fan, Yinghua Lan, Jiwang Chen, Yanxin Huang, Qin Yan, Lisheng Jiang, Shupeng Song, Yongguo Li

**Affiliations:** Department of Infectious Diseases, The First Affiliated Hospital of Harbin Medical University, Post Street 23rd, Nangang District, Harbin, 150001 People’s Republic of China; Department of Infectious Diseases, The Second Hospital of Daqing City, Daqing City, People’s Republic of China

**Keywords:** T-bet transcription factor, Hepatitis B virus, CD8 positive T lymphocytes

## Abstract

**Background:**

The mechanisms leading to virus-specific CD8+ T cell dysfuction in chronic hepatitis B virus (HBV) infection remain to be elucidated. Our study focused on the role of transcription factor T-bet in HBV infection because it is a crucial regulator of T cell immunity.

**Methods:**

We assessed the expression of T-bet along with PD-1, IFN-γ and perforin, in HBV-specific CD8+ T cells from resolved acute hepatitis B (rAHB) patients, chronic hepatitis B (CHB) patients, as well as asymptomatic HBV carriers (ASCs). We observed dynamic changes of T-bet, PD-1, IFN-γ and perforin in acute stage and recovery stage of acute hepatitis B (AHB).

**Results:**

Comparing with other cohorts, HBV-specific CD8+ T cells from rAHB demonstrated a superior ability in T-bet, IFN-γ and perforin expression, but an inferior ability in PD-1 expression. In the CHB group, the level of T-bet has a linear relationship with the level of PD-1, IFN-γ and HBV DNA, respectively. A lower expression of T-bet and PD-1 was observed in ASCs when compared with CHB. A higher expression of T-bet, PD-1, IFN-r and perforin was observed in acute stage when compared with the recovery stage of AHB.

**Conclusions:**

Our results suggest that expression of T-bet may influence the function of HBV-specific CD8+ T cells and thus can be an attractive target for modulation to improve HBV-specific immunity in CHB.

## Background

Both the quantity and quality of the adaptive antiviral immune response influence the clinical outcome of HBV infection [1]. A multi-specific and vigorous T cell response is present in acute hepatitis B (AHB) patients who have successfully cleared HBV infection. However, in chronic HBV infection including CHB and ASCs, the T cell immune responses are weak and oligoclonal [2]. HBV-specific CD8+ T cells play important roles in clearance of HBV infection and in the control of HBV replication [3]. A sustained and potent CD8+ T cell response to HBV antigen is associated with resolved acute HBV infection, but not with chronic HBV infection [4].

Chronic HBV infection acquired perinatally or in early childhood is believed to progress through distinct phases: immune tolerant, immune active and inactive carrier. ASC means a patient is in immune tolerant phase, while CHB means a patient is in immune active phase [5]. The constant existence of viral antigens intrinsic in chronic infection may lead to loss of function in antigen-specific T cells, decreased production of interleukin- 2 (IL-2), tumor necrosis factor (TNF), interferon gamma (IFN-γ) and chemotactic factor β, and gradually impaired proliferation, decreasing cytotoxicity as well as diminishing ability of T cell survival which eventually result in T cell exhaustion [6].

Immunoregulation is centrally involved in T cell exhaustion. These negative pathways can be grouped into three main categories: cell surface inhibitory receptors (such as PD-1), soluble factors (such as IL-10), and immunoregulatory cell types (such as regulatory T cells). In addition, several specific transcriptional pathways have been implicated in T cell exhaustion. Particularly, the transcription factor T-bet is centrally involved in CD8+ T cell exhaustion [7].

T-bet, which belongs to the T-box transcription factor family, is encoded by the Tbx21 gene and expressed in many immune cells, showing an extensive immunoregulatory function [8]. T-bet sustains the effector function of CD8+ T cells through various mechanisms, such as regulation of CD8+ T cell proliferation, suppressed expression of inhibitory receptors such as PD-1, and promotion of IFN-γ and perforin secretion [9]. Dysfunction of CD8+ T cells has been found in mice with defective Tbx21 and chronic infection occurred after LCMV infection [10]. Chronic HIV infection leads to decreased T-bet expression in HIV-specific CD8+ T cells [11, 12]. Moreover, T-bet has been implicated as an anti-tumor regulator and pathogenic factor for autoimmune diseases [13, 14].

Currently, the correlation between the impaired regulation of T-bet and the development of chronic HBV infection has not been established. A study by Kurktschiev PD et al. assessed the influence of T-bet on CD8 + T cells activity observed during acute and chronic HBV infection [15]. In our study, patients with HBV infection were further divided into three groups: AHB, CHB and ASCs. We have determined T-bet expression in these groups and examined its correlation with the clinical outcome. Our observations suggest a central role of T-bet in regulating different immune states of HBV infection.

## Methods

### Research subjects

From May 2013 to December 2014, patients and controls with positive human leukocyte antigen-A2 (HLA-A2) from the Department of Infectious Diseases in the First Affiliated Hospital of Harbin Medical University (Harbin, China) or the Department of Infectious Diseases in the Second Hospital of Daqing (Daqing, China) were recruited for this study. Among these volunteers, there were 9 AHB patients, 18 CHB patients, and 15 ASCs. The patient characteristics are summarized in Table 1.

**Table 1:** Clinical characteristics of the three patient groups

	AHB *n =* 9	CHB *n =* 18	ASCs *n =* 15	*P* values
Age (years, mean ± SD)	33.67 ± 5.32	42.45 ± 4.67	20.22 ± 3.71	<0.0001
Gender ( male/female)	5/4	10/8	8/7	
HBsAg (+/−)	9/0	18/0	15/0	
HBsAb (+/−)	1/8	0/18	0/15	
HBeAg (+/−)	3/6	11/7	15/0	
HBeAb (+/−)	8/1	7/11	0/15	
HBcAb (+/−)	9/0	18/0	15/0	
Median HBV DNA level (log_10_ IU/mL)	4.69(2.69–5.55)	5.31(2.26–8.58)	7.99(4.04–8.57)	=0.0012
ALT (units/L)mean ± SD	1773.11 ± 1038.47	523 ± 775.40	19.43 ± 5.47	<0.0001

Abbreviations: *AHB* acute hepatitis B, *CHB* chronic hepatitis B, *ASCs* asymptomatic hepatitis B virus carriers, *HBsAg* hepatitis B virus surface antigen, *HBsAb* HBsAg antibody, *HBeAg* hepatitis B e antigen, *HBeAb* HBeAg antibody, *HBcAb* hepatitis B core antibody, *HBV DNA* hepatitis B virus DNA, *ALT* alanine aminotransferase. *P* values given as comparison among 3 groups by Kruskal-Wallis test

### Diagnostic criteria

Subjects were selected according to previously described criteria [16] as the following: AHB was defined as acute onset of nonspecific flu-like symptoms and jaundice in previously healthy persons with peak alanine aminotransferase (ALT) elevation 10 times above the upper limit of normal, and was confirmed by concomitant detection of hepatitis B surface antigen (HBsAg), HBV DNA, or anti-hepatitis B core IgM antibody (anti-HBc-IgM). rAHB was confirmed by seroconversion of hepatitis B surface antibodies (anti-HBs). CHB was defined by detection of HBV DNA or HBsAg for more than 6 months with ALT fluctuations. ASCs were defined as HBsAg positive, ALT and aspartate aminotransferase (AST) within the normal range for more than 3 visits, and a history of HBV infection. Patients with other possible causes for chronic liver damage, such as alcohol use, drug use, congestive heart failure and autoimmune diseases, and pregnant women were also excluded from this study.

### Ethics statement

The experiments in this study were carried out under the guidance of moral standards described in Declaration of Helsinki and International Ethical Guidelines for Biomedical Research Involving Human Subjects by Council for International Organizations of Medical Sciences (CIOMS), with the approval of ethics committee in First Affiliated Hospital of Harbin Medical University (approval ID: ChiCTR-CCC-14004949). An informed consent was signed by all study subjects.

### Synthetic peptides, pentamers, and cytokines

Recombinant HBV core antigen (HBcAg) covering the overall protein sequence of HBV genotype D was purchased from ProSpec (NJ, USA). Human leukocyte antigen (HLA) restricted peptide HBV core antigen 18–27 (FLPSDFFPSV and FLPSDFFPSI, HBV c 18–27) was purchased from Proimmune (Oxford, UK). HBcAg and HBV c 18–27 were used for the in vitro stimulation of HBV-specific CD8+ T cells. HBV c 18–27 was detected by PE-labeled MHC-I restricted pentamers (Proimmune, Oxford, UK). Recombinant human IL-2 (PeproTech, NJ, USA) was used for stimulation experiments.

### Monoclonal antibodies for flow cytometry

FITC anti-human HLA-A2 (BioLegend, San Diego, CA, USA), PE-Cy7 anti-human/mouse T-bet (eBioscience, San Diego, CA, USA), APC anti-Human CD8a (eBioscience, San Diego, CA, USA), FITC anti-human CD279 (PD-1) (BioLegend, San Diego, CA, USA), PerCP anti-CD14 (eBioscience, San Diego, CA, USA), APC-eFluor®780 anti-CD19 (eBioscience, San Diego, CA, USA) and 7-AAD (BD Biosciences, San Diego, CA, USA) were used for flow cytometry. Isotype control was used for each antibody. The Foxp3/Transcription Factor Staining Buffer Set Kit (eBioscience, San Diego, CA, USA) was used for intracellular staining according to the manufacturer’s instructions.

### HLA-A2 genotype detection

Screening for HLA-A2 was performed by staining peripheral blood mononuclear cells (PBMCs) with a FITC-labeled mouse anti-HLA-A 2 and isotype control (BD, Biosciences, San Diego, CA, USA).

### Isolation of PBMCs

PBMCs were isolated from fresh heparinized blood using Ficoll-Hypaque density gradient centrifugation and were either analyzed directly or resuspended in medium for stimulation of PBMCs.

### PBMC stimulation

For HBV-specific CD8+ T cells expansion, PBMCs were cultured for 10 days in RPMI 1640 medium containing 2 mM l-glutamine, 1 mM sodium pyruvate, 100 U/ml of penicillin, 100 μg/ml of streptomycin and 5 % human type AB serum. PBMCs were seeded at a density of 1 × 10^6^/ml in 24-well plates, and 1 ml medium was used in each well. In the cytokine-stimulated groups, IL-2 (20 IU/ml) was added on day 0. The antigen-stimulated groups received 5 μg/ml of antigen on day 0 and were re-stimulated with the same dose of antigen on day 10. HBcAg (5ug/ml;) and HBV c 18–27 (FLPSDFFPSV, 5ug/ml; FLPSDFFPSI, 5ug/ml) were used for stimulation. After stimulation, cells were prepared for flow cytometry by cell surface staining and intracellular staining [17].

### Cell surface staining and intracellular staining for flow cytometry

2–3 × 10^6^ PBMCs were stained with MHC-I pentamers according to the manufacturer’s instructions. After staining with viability dyes and antibodies specific for surface markers, cells were fixed with intracellular fixation buffer and permeabilized with permeabilization buffer. After the permeabilization step, cells were stained with intracellular markers. For ex vivo staining, at least 200,000 PBMCs were collected for pentamer + CD8+ T cells as they were low frequency cells. Samples were acquired on a FACSCanto II flow cytometer (BD, Biosciences, San Diego, CA, USA). Data were analyzed with FlowJo 9.6.1 software (Tree Star). Gating strategy excluded monocytes (CD14+), B-lymphocytes (CD19+), and dead cells (7-AAD+) by a dump channel.

### Enzyme-linked immunosorbent assay (ELISA)

PBMCs were cultured for HBV-specific CD8+ T cell expansion with stimulation by HBV antigen and peptides for 10 days. Cells were then seeded into 96-well plates at a density of 1 × 10^5^ cells in 100 μl RPMI 1640 medium in each well, and then stimulated again with HBV c 18–27 for 18 h. Subsequently, the cells and supernatant were collected. A human IFN-γ embedded ELISA kit (Dakewe Biotech, Beijing, China) and a human perforin embedded ELISA kit (Dakewe Biotech) were used according to the manufacturer’s instructions. Data were detected with a microplate reader and analyzed thereafter.

### Statistical analysis

All data were analyzed using Prism 5.0 software (GraphPad, CA, USA). Data were expressed as the median with range or mean ± SEM. The Kruskal-Wallis Test was performed to test the differences between the three groups, and the difference of every two groups was tested using the Mann–Whitney test (median) or *t* test (mean). Spearman’s rank correlation test was used for correlation analysis. *P*-values of *P* < 0.05 were considered significant.

## Results

### HBV-specific CD8+ T cell responses

**Fig. 1:**
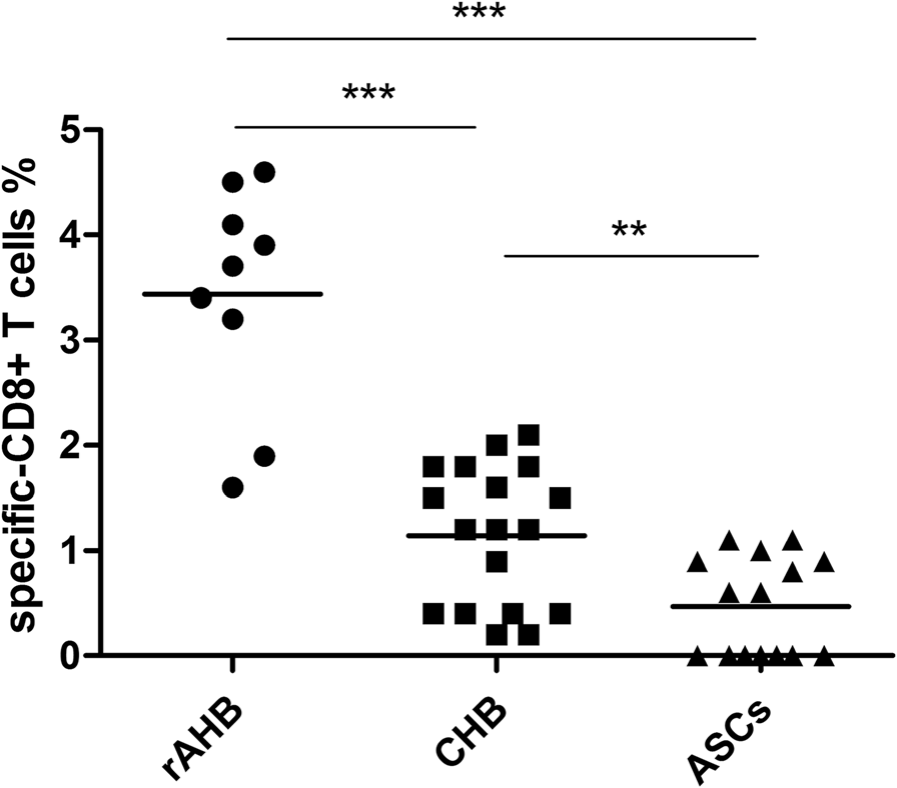
Percentage of pentamer + CD8+ T cells in the total CD8 + T cells after *in vitro* induction. After induced amplification, peripheral blood mononuclear cells (PBMCs) from patients with resolved acute hepatitis B (rAHB), chronic hepatitis B (CHB) and asymptomatic hepatitis B virus carriers (ASCs) were used to detect the percentage of pentamer + CD8+ T cells in total CD8 + T cells. Each dot represents an individual data point and the horizontal lines represent the mean percentage of pentamer + CD8 T cells in the total CD8+ T cells determined by flow cytometry. **represent *P* < 0.01,***represent *P* < 0.001, by Mann Whitney test

Cell surface staining of PBMCs after *in vitro* induction was carried out, and the percentage of HBV-specific CD8+ T cells was analyzed by FACS for patients in the rAHB, CHB or ASC groups respectively. The percentage of HBV-specific CD8+ T cells in the total CD8+ T cells of the rAHB, CHB and ASC group was 2.04 %, 1.14 % and 0.46 %, respectively (*P <* 0.0001 by Kruskal-Wallis test, Fig. 1).

### T-bet expression level in HBV-specific CD8+ T cells

**Fig. 2:**
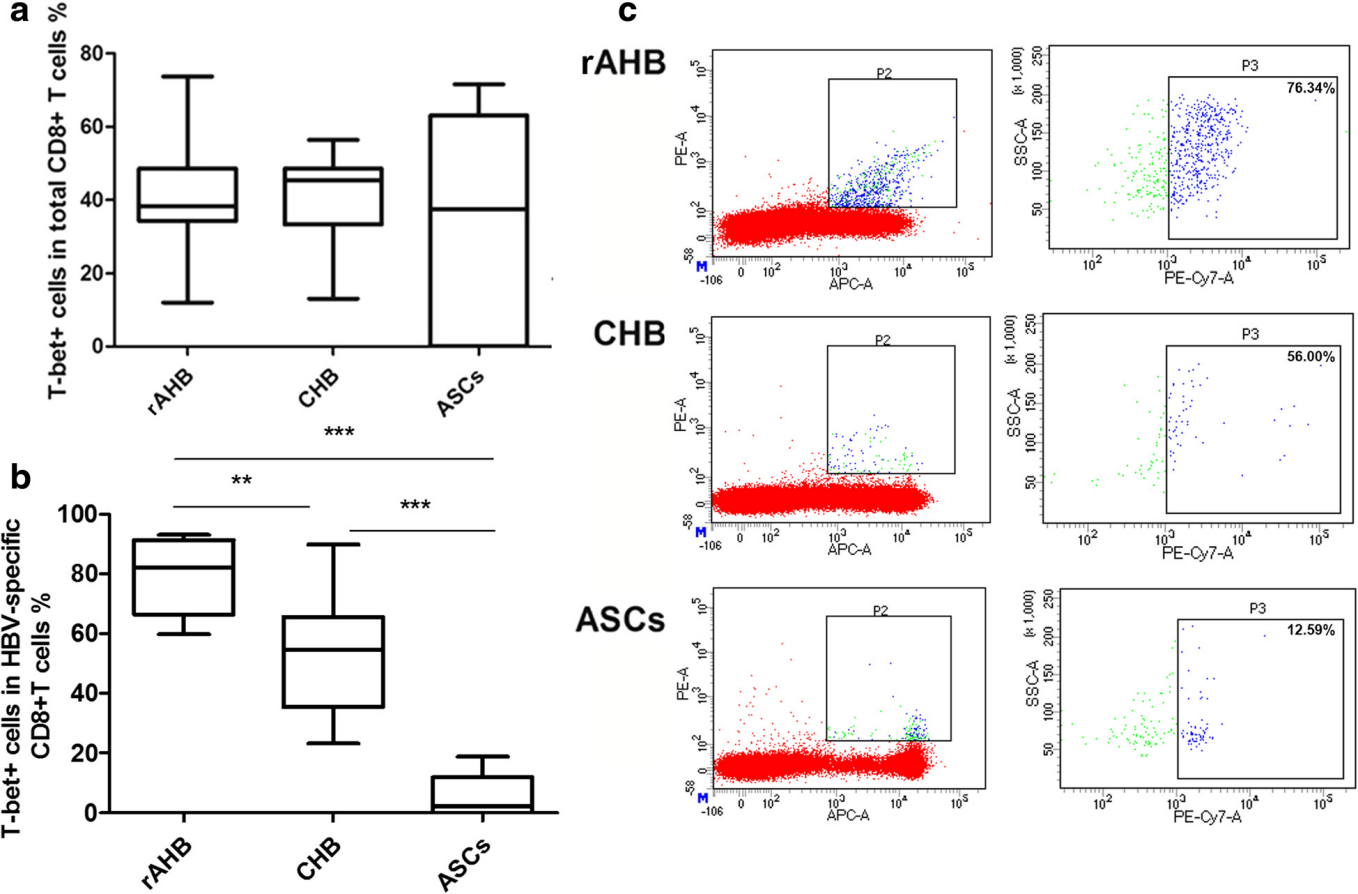
T-bet expression in HBV-specific CD8+ T cells. **a** Percentage of T-bet + cells in the total CD8+ T cell population (CD8 + T-bet+/CD8+) determined by flow cytometry. Data are shown as median (IQR). IQR = interquartile range. *P >* 0.05, by Kruskal-Wallis H test. **b** Percentage of T-bet + cells in HBV-specific CD8+ T cells (pentamer + CD8 + T-bet+/pentamer + CD8+) determined by flow cytometry. Data are shown as median (IQR). IQR = interquartile range. **represent *P* < 0.01,***represent *P* < 0.001, by Mann Whitney test. **c** Representative flow cytometry data of T-bet expression

The percentage of T-bet + cells in the total CD8+ T cells of the rAHB , CHB and ASC groups was 41.21 %, 39.94 % and 31.42 %, respectively, with no significant difference (*P* = 0.8619, by Kruskal-Wallis test, Fig. 2a). As shown in Fig. 2b, the percentage of T-bet + cells in the HBV-specific CD8+ T cells of the rAHB, CHB, and ASC groups was 78.62 %, 52.67 % and 10.37 %, respectively (*P <* 0.0001, by Kruskal-Wallis H test). The percentage in the rAHB group was higher than that in CHB group (*P* < 0.01, by Wilcoxon test), while the latter was higher than that in the ASC group (*P* < 0.01, by Wilcoxon test).

### PD-1 expression level in HBV-specific CD8+ T cells

**Fig. 3:**
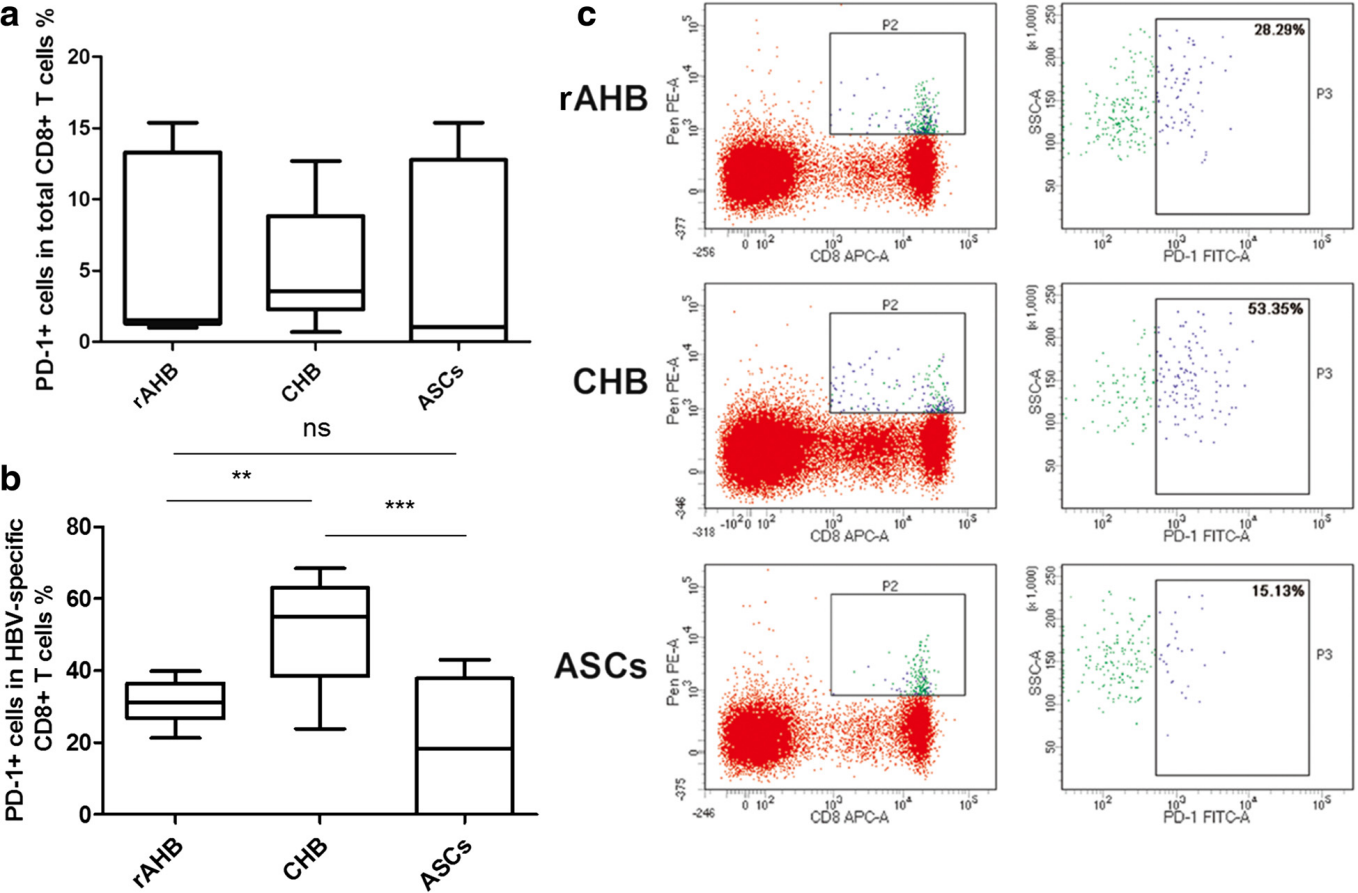
PD-1 expression in the HBV-specific CD8+ T cells. **a** Percentage of PD-1+ cells in total CD8+ T cells (CD8 + PD-1+/CD8+) determined by flow cytometry. Data are shown as median (IQR). IQR = interquartile range. *P >* 0.05, by Kruskal-Wallis H test. **b** Percentage of PD-1+ cells in the HBV-specific CD8+ T cell population (pentamer + CD8 + PD-1+/pentamer + CD8+) determined by flow cytometry. Data are shown as median (IQR). IQR = interquartile range. ns represent no significant, **represent *P* < 0.01, ***represent *P* < 0.001, by Mann Whitney test. **c** Representative flow cytometry data of PD-1 expression

The percentage of PD-1+ cells in the total CD8+ T cells of the rAHB, CHB and ASCs was 6.79 %, 5.64 % and 7.75 %, respectively, with no significant difference (*P* = 0.8099, by Kruskal-Wallis test, Fig. 3a). The percentage of PD-1+ cells in the HBV-specific CD8+ T cells of the rAHB, CHB and ASC groups was 31.51 %, 50.68 % and 17.44 %, respectively, with significant difference (*P* = 0.0013, by Kruskal-Wallis test, Fig. 3b). The percentage in the rAHB group was lower than that in the CHB group (*P* < 0.01, by Wilcoxon test). The percentage in the CHB group was higher than that in the ASC group (*P* < 0.01, by Wilcoxon test).

### Induced IFN-γ and perforin levels in HBV-specific T cells

**Fig. 4:**
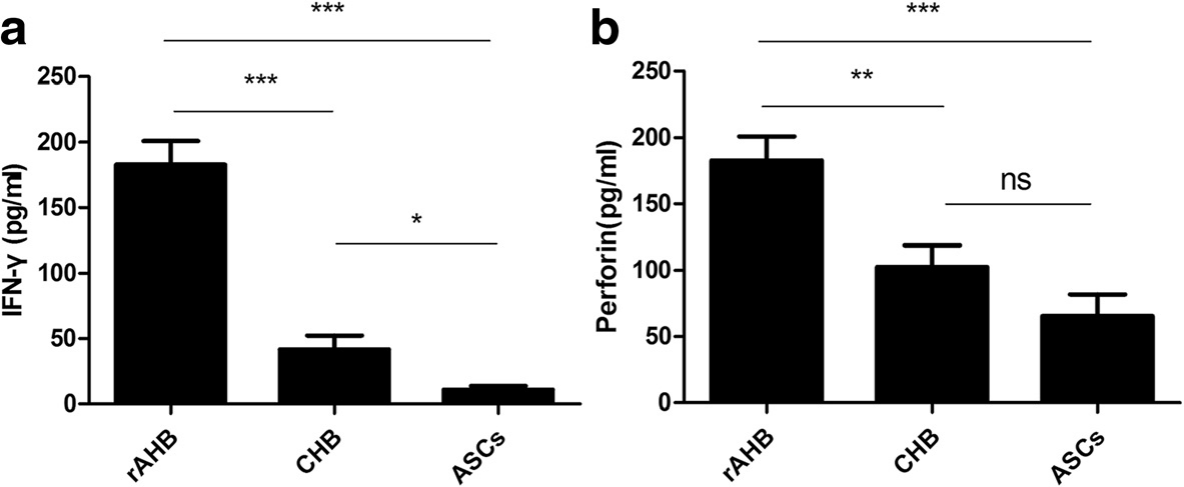
IFN-γ and perforin levels in the HBV-specific CD8+ T cell population. **a** Mean level of IFN-γ produced by HBV-specific T cells. Error bars indicate SEM. **b** Mean level of Perforin levels produced by HBV-specific T cells. Error bars indicate SEM. *represents *P <* 0.05, **represents *P <* 0.01, ***represents *P <* 0.001, ns means no significant, by unpaired t test

PBMCs isolated from the three groups were induced for 10 days with HBV antigens, followed by a further stimulation with HBV antigens for 18 h. IFN-γ and perforin in the supernatant were detected by ELISA. PHA-stimulated (5ug/ml) samples were used as positive controls, and samples without antigen stimulation were used as negative controls. Virus antigen stimulation resulted in similar IFN-γ and perforin levels compared with that stimulated by PHA. The IFN-γ levels in the AHB group were higher than those in the CHB group (*P* < 0.0001, by unpaired t test) and those in the ASC group (*P* < 0.0001 by unpaired t test). The IFN-γ levels in the CHB group and the ASC group had no significant differences (*P* = 0.2011, by unpaired t test). The perforin levels in the rAHB group were higher than those in the CHB group (*P* < 0.0001, by unpaired t test) and those in the ASC group (*P* < 0.0001, by unpaired t test). The perforin levels in the CHB and ASC groups had no significant difference (*P* = 0.1842, by unpaired t test, Fig. 4).

### Relationships between T-bet, PD-1, IFN-γ and HBV DNA in CHB

**Fig. 5:**
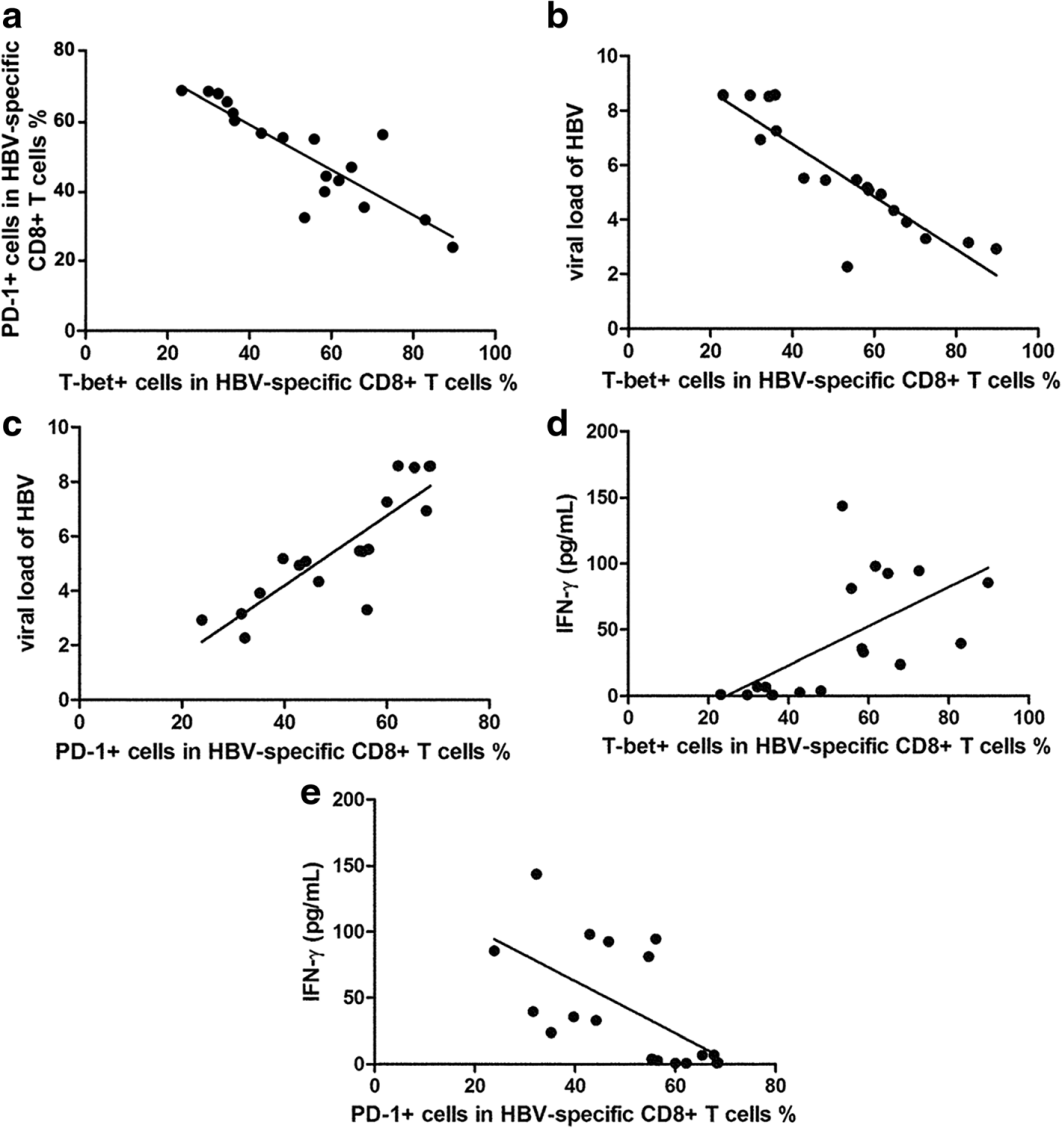
Correlation between percentage of T-bet + or PD-1+ HBV-specific CD8+ T cells and HBV DNA content or IFN-γ level in PBMC from CHB patients. **a** The percentage of T-bet + cells was negatively related to the percentage of PD-1+ cells (r = −0.8638, *p <* 0.0001); **b** The percentage of T-bet + cells was negatively related to HBV DNA (r = −0.8741, *p <* 0.0001); **c** The percentage of PD-1+ cells was positively related to HBV DNA (r = 0.8864, *p <* 0.0001); **d** The percentage of T-bet + cells was positively related to IFN-γ level (r = 0.7193, *p =* 0.0008); **e** The percentage of PD-1+ cells was negatively related to IFN-γ level (r = −0.7027, *p =* 0.0011), Spearman rank correlation test was used for data analysis

To further explore if T-bet can suppress PD-1 expression, promote IFN-γ production and eventually affect serum HBV DNA levels, we analyzed the correlation between the percentage of T-bet + HBV specific CD8+ T cells and PD-1+ HBV-specific CD8+ T cells, HBV DNA load or IFN-γ level of the CHB patients. As shown in Fig. 5, the percentage of T-bet + HBV-specific CD8+ T cells showed a significant negative correlation to the percentage of PD-1+ HBV-specific CD8+ T cells (r = −0.8638, *P* < 0.0001; Fig. 5a). The percentage of T-bet + HBV-specific CD8+ T cells showed a significant negative correlation to viral load of HBV (r = −0.8741, *P* < 0.0001; Fig. 5b). The percentage of PD-1+ HBV-specific CD8+ T cells showed a significant positive correlation to viral load of HBV (r = 0.8864, *P* < 0.0001; Fig. 5c). The percentage of T-bet + HBV-specific CD8+ T cells showed a significant positive correlation to IFN-γ level (r = 0.7193, *P* = 0.0008; Fig. 5d), The percentage of PD-1+ HBV-specific CD8+ T cells showed a significant negative correlation to IFN-γ level (r = −0.7027, *P* = 0.0011; Fig. 5e)

### Dynamic changes of T-bet, PD-1, IFN-γ and perforin in AHB

**Fig. 6:**
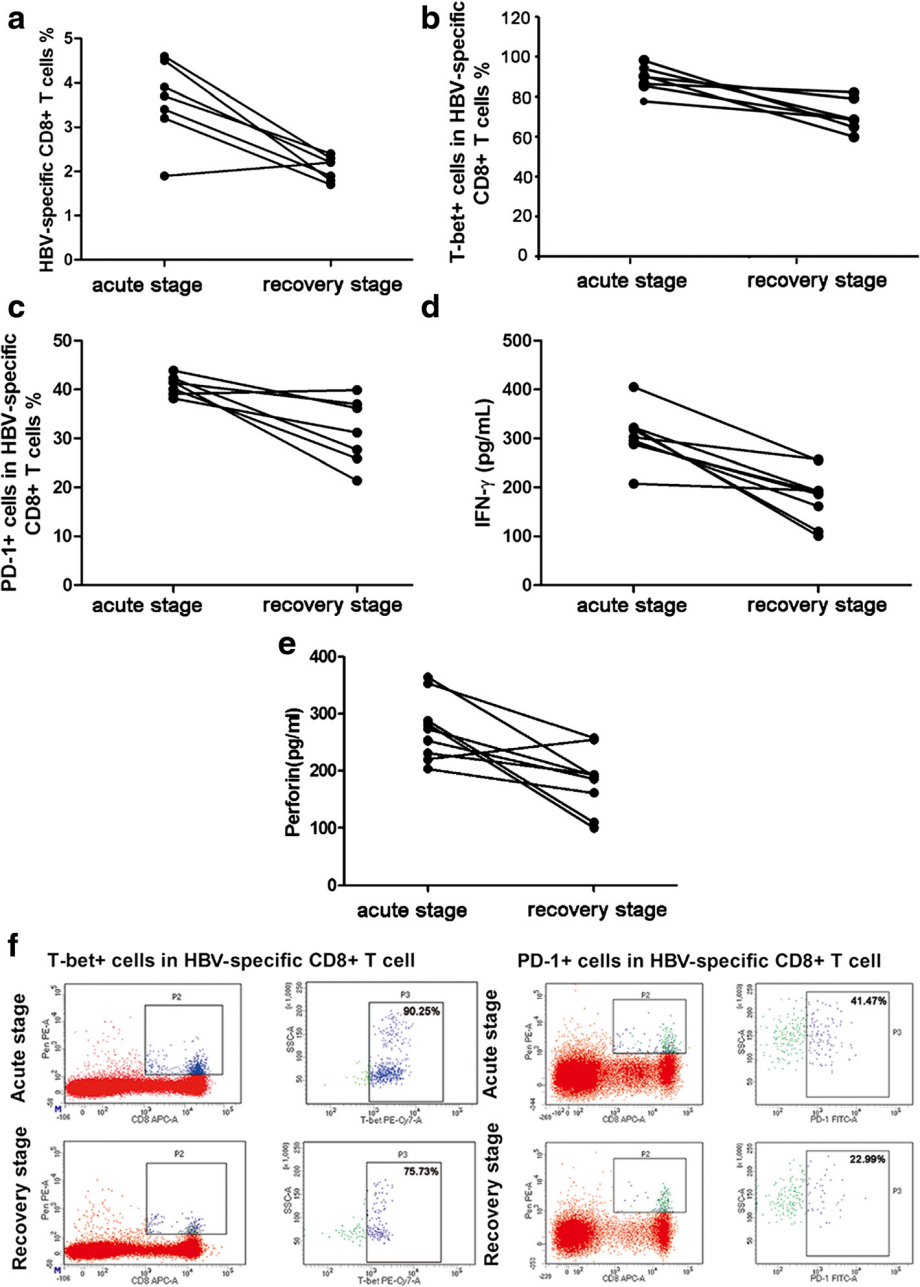
Changes of HBV-specific CD8+ T cells, T-bet, PD-1, IFN-γand perforin in AHB. **a** Percentage of HBV-specific CD8+ T cells existed in the acute and recovery stages (*p =* 0.0343, by Wilcoxon signed rank test); **b** Level of T-bet in HBV-specific CD8+ T cells in the acute and recovery stages (*p =* 0.0156, by Wilcoxon signed rank test); **c** Level of PD-1 in HBV-specific CD8+ T cells in the acute and recovery stages (*P =* 0.0313, by Wilcoxon signed rank test); **d** Level of IFN-γ in HBV-specific CD8+ T cells in the acute and recovery stages (*P =* 0.0006, by paired t test); **e** Level of perforin in HBV-specific CD8+ T cells in the acute and recovery stages (*P =* 0.0062, by paired t test); **f** Representative flow cytometry data of T-bet and PD-1 expression in acute stage and recovery stage of AHB

We further compared the percentage of T-bet + cells and PD-1+ cells in the total HBV-specific CD8+ T cells, as well as their capability for secretion of IFN-γ and perforin between the acute stage and recovery stage of the 7 AHB patients. Peripheral blood was collected when the patients had been in hospital for 2 weeks, or had left hospital for 16 weeks, respectively. The cells were induced by antigens and each index was detected after a 10-day *in vitro* culture. As shown in Fig. 6, along with the recovery of disease, expression of T-bet, PD-1, IFN-γ and perforin decreased 4 months after hospital discharge accompanied by reduced percentage of HBV-specific CD8+ T cells.

## Discussion

The poor T-bet expression in virus-specific CD8+ T cells may be a central regulation factor for T cell exhaustion [7]. We compared *in vitro* amplification of HBV-specific CD8+ T cells isolated from rAHB and CHB patients and ASCs, under stimulation by viral antigens. Similar to previous study [4], we found dysfunction and low response of HBV-specific CD8+ T cells in the CHB patients and ASC patients comparing with those of rAHB patients. The expression of T-bet in the HBV-specific CD8+ T cells of CHB and ASCs were significantly lower than those of rAHB patients. The T-bet expression was lowest in the HBV-specific CD8+ T cells of ASCs. It has been demonstrated that overexpression of T-bet can promote T cell response, while suppression of T-bet expression can inhibit T cell response [18]. Our data showed expression of T-bet was in accordance with HBV specific CD8+ T cell response. Similar results concerning T-bet expression were observed in HIV infected patients [11]. Our data suggest that T-bet might regulate the level of the HBV-specific CD8+ T cell response, and low T-bet levels might be an important factor leading to exhaustion of cytotoxic T cells in chronic hepatitis B. In the CHB group, the level of T-bet had a negative correlation with the level of HBV DNA, suggesting T-bet expression is vital for HBV clearance. The study by Kurktschiev PD et al. compared T-bet expression between AHB and CHB [15]. Our study further showed that the level of T-bet was lower in ASCs than in CHB. We propose that lower expression of T-bet may be a factor of immune tolerance and higher expression of T-bet may trigger a biological process leading to loss of immune tolerance.

The axis of PD-1 and its ligand is a major inhibitory receptor pathway involved in CD8+ T cell exhaustion [7], and PD-1 can be suppressed by T-bet in chronic murine LCMV infection [9]. IL-12 can induce T-bet and decrease PD-1 expression level in chronic hepatitis B and restore IFN production [19]. In our study, PD-1 expression was higher in the HBV-specific CD8+ T cells of CHB patients comparing with those of rAHB patients, and a higher PD-1 expression was coexisting with a lower HBV-specific CD8 + T cell response. The PD-1 expression level was positively correlating with the level of HBV DNA in CHB patients. This suggests that PD-1/PD-L1 pathway may inhibit HBV clearance by inhibiting HBV-specific CD8+ T cells. A similar PD-1 expression was observed in HIV infected patients [20]. It was noted that the PD-1 expression was lower in the ASC group than in the CHB group. One reason is that ASC patients is in immune tolerant phase, and PD-1 expression is lower in immune tolerant phase than in immune active phase [21]. Another reason is that the ASC patients we chose were younger than the CHB patients, and PD-1 expression is lower in the young adult patients than in the older cohort from chronic HBV infected patients [22].

As a ‘master regulator’ of cell-mediated immunity, T-bet participates in regulation of genes encoding effector molecules in immune cells, such as IFN-γ, perforin, and granzymes [23]. CD8+ T cells participate in clearance of intracellular viruses upon production of IFN-γ, and can lyse target cells with perforin and granzymes [24]. We observed a high level expression of IFN-γ and perforin in the rAHB group and a low level expression of these genes in the CHB and ASC groups, with a positive correlation between T-bet and IFN-γ production in the CHB group. These results indicate that low T-bet expression in chronic HBV infection might lead to impaired production of IFN-γ and perforin. Because the transcription factor eomesodermin also controls production of IFN-γ and peforin in effector CD8 + T cells, further studies of the roles of eomesodermin in chronic HBV infection is necessary [25].

We followed up the AHB patients to determine differences in the acute stage and recovery stage of AHB. Our results showed that both T-bet and PD-1 had higher levels along with IFN-γ and perforin in the acute stage than in the recovery stage. A previous study also indicated that at clinical onset of acute HBV infection, PD-1 was significantly up-regulated and subsequently led to the functional suppression of HBV-specific CD8+ T cells, and that following disease resolution, HBV-specific effector CD8+ T cells developed into memory T cells. During this period, the dynamic PD-1 decrease was numerically correlated with the reduction of HBV-specific CD8+ T cell frequency [26]. Another study showed that the effect of T-bet on PD-1 expression was modest during acute infection but became greater during chronic infection [9]. We propose that in the acute stage, a high level of T-bet expression can promote amplification of CD8+ T cells and result in virus clearance, and that a high level of PD-1 might lead to rapid apoptosis of effector cells. So, the balance between T-bet and PD-1 in the acute phase is critical to virus clearance and pathological damage control.

Concerning the potential mechanism underlying low expression of T-bet in virus specific CD8+ T cells in patients with chronic HBV infection, Smith et al. suggested that during constant infection, antigen presenting cells might influence the transcription of transcription factors in virus specific T cells, such as T-bet [27]. EJ Wherry et al. suggested that constant virus infection led to a continuous conversion from T-bet^high^ progenitor cells to Eomes^high^ cells in virus specific CD8+ T cells, which eventually led to the disappearance of T-bet^high^ cells [28]. Previous studies have shown that inflammatory signals (e.g., IL-12) not only enhance T-bet expression but may also repress Eomes and thus play a dominant role in regulating memory/effector T-cell potential [29]. The IL-21–induced cytotoxic T cell (CTL) function is T-bet dependent, because T-bet deficiency results in defective IL-21–dependent cytotoxicity in CD8+ T cells in vitro and in vivo [30]. Therefore, it is possible that lack of IL-12 or IL-21 results in low T-bet expression in chronic hepatitis B.

## Conclusion

The expression of T-bet is closely related to exhaustion of T cells in chronic hepatitis B, and T-bet might be a critical factor leading to chronic HBV infection. Further understanding of T-bet function may provide new therapeutic approaches for chronic hepatitis B. We observed different levels of T-bet and PD-1 expression between CHB and ASC patients, and these may explain the different immune status during chronic HBV infection.
